# Supplementary material for: “My mother in-law forced my husband to divorce me”: Experiences of women with infertility in Zamfara State of Nigeria
Source: PLoS One. 2019 Dec 19;14(12):e0225149. doi: 10.1371/journal.pone.0225149 (PMC6922459; doi:10.1371/journal.pone.0225149)
Supplement: S1 Transcript — (DOCX) [file pone.0225149.s001.docx]

RESPONDENT1 TRANSCRIPT

BIO DATA

Q. Can you tell me about you?

I will be around 26 years now; I am married woman and got married since I was young. I am Muslim by religion. I attended primary and islamiyya schools. I sell rice and I am Hausa by tribe. I have no children

Psychological experiences

Can you share with me how you felt when you were told that, you have infertility?

I understood that I was in worries because is something needed by everybody which will be of useful to him. But I am deprived of it due to some problems.

Q. Which problems?

R. The problems were not associated with lower abdominal pains, we lived for many years with my first husband, but we couldn’t have children. He had some other children with his second wife. I got married to my second husband after his death.

1. As a married woman with this condition how have you been feeling deep in you?
2. I felt that I am not in happiness due to lack of children and I found myself in worries and some fear of being alone in the world. I feel more disturbed some other time, because everyone will need to have a child given to him by God which will help me. So if I remembered that I don’t have a child, I will be so disturbed.-
3. what are those things when you see them make you remember and have such disturbances?
4. *(Noding),yes is because* I have relatives who have children, so it makes me remembered and have psychological unrest when we met in any gathering at present of their children, considering that, I am only the one who don’t have child among them.
5. How do you perceive life in this situation?
6. I look at my life as not the same as compared to others.
7. As how?
8. Is because a child makes you happy but I don’t have one, so that is what causes the disturbances

Social experiences

Q. Can you kindly share with me life situation in your matrimonial home about the diagnosis of this problem?

R. Yes I am woman with deficiency, because you live in a house with no children to assist you

Q. Considering our culture which is in full of need for children, what are some of your experiences in relation to your relatives, husband ,his relatives and friends

R. My relationship with them?

Q. Yes .

R. We relate, but you know if you don’t have child some of them use to show differences-

Q. Can you please tell me such differences?

R. Differences because you don’t have a child?

Q. Yes.

R. Ok yes if one of their children came into your house doing something, they will call him back to come out of your house.

Q. What next have you faced?

R. Yes that it is if you don’t have a child, you most face problem with them especially by calling him back.

Q. What about words do you receive bad words from them?

R. Yes some will be indirect, but some will utter some offensive words on you. for instance, there was a time I sent one of their daughter in which she lost what I gave to her, then she came back crying without even knowing she was the one; because I was praying then. So my husband asked her what happened. And then he went out for prayer; I don’t know she was the one crying. So her mother said I sent her away because I didn’t give birth to her. I can`t talk to their children when they did wrong. That episode really hurt me seriously.

Q. What about your relationship with husband?

R. He needs ways of having this problem solved.

Q. Ok, I want know your relationship with him when he knew that you have this problem?

R. Actually he treated me well despite my condition.

Q. As for your relatives what has been your relationship.

R. Yes my friends and relatives support me well, no any stress received from them

Q. Can you please describe how you relate with people before and after the diagnosis?

R. Actually compared to previous time I am more worried now than before, because I don’t have children.

Q. From your understanding of the situation, how will you compare your position in the society before and after the diagnosis?

R. I still relate well with people despite my condition. My position in society hasn’t change yet

**Coping strategies**

Q. Looking at all that you have shared with me, have you been using some measures to adjust? R. (Smile) I don’t use any way rather than praying to God to give me child, even if I am disturbed I will leave things to God to ease them for me.

Q. Any other ways?

R. Beside this?

Q. Yes.

R. Like going to seek for medicine?

Q. Yes like one can use other ways for instance adoption and rest.

R. Yes is true (high voice) the use of adoption? Yes I adopted a child previously in order to assist me.

Q. what about now?

R. Presently I only invite children of my relatives and that of friends to come to my house frequently. There is one my sister`s child that use to come to my house but not to stay with me permanently

**Health seeking behaviour**

Q. Can you share with me general situation regarding your seeking for help?

**R.** Like going to the hospital? Actually previously I didn’t seek for any help anywhere, till of recent I went to the hospital and sought for help. I undergone some medical examinations and my problem was confirmed

Q. What are the factors that make you to come to hospital seeking for help?

**R.** Yes it was my husband that said I should go to the hospital

Q. Anything you want share with me?

R. Do you mean related to the questions you asked? Ok I thing that is all I can say for now
