# Supplementary material for: “My mother in-law forced my husband to divorce me”: Experiences of women with infertility in Zamfara State of Nigeria
Source: PLoS One. 2019 Dec 19;14(12):e0225149. doi: 10.1371/journal.pone.0225149 (PMC6922459; doi:10.1371/journal.pone.0225149)
Supplement: S2 Transcript — (DOCX) [file pone.0225149.s002.docx]

Respondent2

As I explained to you, my name is Yakubu Lawali a masters student with university of Ghana, legon. I am conducting my research on psychosocial experiences of women with infertility and their coping strategies in Zamfara.

Q. can you tell me little about you

R. I am 28 years old. I attended islamiyya that is I got the ability to read and write but I didn’t attend even a primary school because our father refuse female to attend to such schools. I have 3 children. I sell nothing.

Q. we are moving to psychological experiences. When you are told that, you have infertility how did you feel?

R. I actually faced problem because I was so disturbed. This is because of what has been happening in my matrimonial home. My husband`s relative have been saying a lot of things. So I must be angry and disturbed; my heart beats a lot because of this. I have been thinking of what brought about these things why are they against me? I didn’t use any form of family planning,in fact, it was even brought to me I said no because my husband need children, this is a test from God that is why I am so surprised. Has it been I used family planning I will have disturbed more; it was brought to me in my room I swear to God i told them I wouldn’t use it. I look at it that, has it been I used it I will suffer more disturbances and sorrow. So that is what is in my mind. As I am talking to you my heart is hurting me; as I touch here (epigastric region) It will pain me and that is because of sorrowful events I passed through

Q. Sorrowful events?

R. Yes sorrow, because my mother in law put pressure on my husband; I shade tears everyday; is very had to spend a week without shedding tears. You see is all part of my problem since I leave with my husband peacefully until when this thing happened to me (infertility) and what I faced from them

Q. you are married woman and considering our culture full of need for children and now you have this problem how do you feel?

R. I feel that I need children because my husband needs them. My husband loves my children and that of my mate. I love to have more. Before we were in serious poverty but now we are out of it and I want give birth during this period (wealthy) phone ringing.

Q yes I can hear you

R. yes I said before we were In serious poverty because I lived in my husband`s family house. When he became rich they started putting pressures on me complaining that, how will I stay in that house alone? How will I be in that house (their new house) alone? So when they couldn’t succeed they used this opportunity of my infertility to tell him to marry another wife because he needs to have more children; that is what they have been doing. So they used the fact that, he needs children to succeed on their mission. They got a lady in my neighborhood and made him to marry her and we are still living together with her. You see it (infertility) is the root of all these. Because has it been I am well all these things wouldn’t had happened

Q. what reminds you of your infertility?

R. My problem?

Q. yes your infertility issues are there things when you hear or see them make you remember?

R. Yes, I have them. Like mocking or showing some injustice to me; I must be angry because my infertility is the cause of everything I am in now. My husband needs children and my mate gives birth for him, so he must show some level of closeness to her than me. Whenever she delivered he will spend money for her which I couldn’t have such opportunity before, because there was no money by then. It is now that he is wealthy and it is the time they are jealous of me; they want me out of the house (divorce). He told them that, he will not divorce me but he will marry another wife. You see it is the root of my problem. When I remembered of our lives previously my heart palpitates, I have to hold my chest followed by tears. It is very difficult to spend a week without shading tears. Sometime even if I am praying I will remember our lives before. In our school nobody agreed to marry him but I agreed and now it reached to a time when his mummy will say that he should marry another wife. The worse part of it is that, the lady is my neighbour she uses to carry my children; I don’t even know about the wedding till when I was told to come and take some clothes for the lady close to the wedding day. So this must put one in a sorrow. This is an injustice, you lived with him for 2 years no food and now just because of this (silent and tears). Now just because of this problem they are telling me to leave the house forgetting that it was together with me he became wealthy. She even collected my children and said to me that I should leave the house. He (my husband) begged her and she allowed me then. She was in sokoto when we suffered. No food, no clothes and during sallah (eid period) it was my parent that sent cloth for me to celebrate eid. I told nobody beside you. I sold all my room materials for him, I never tell anybody. When I delivered I didn’t mind the small things he gave to me. Whether I eat or not I didn’t mind; I spent many days without eating and I used to fast. I know it was a test from God. He travelled to village and spent long time I didn’t mind. But doesn’t the time change? Mother in law because her son is now rich wants a woman who suffered with him to leave the house!! She said that he should divorce me and he said no if it is because of infertility, then she said she knew what to do. That was the reason why she gave him a neighbour to marry. Her mother (of the lady he married) used to come to my house and discuss some issues!! We had very good understanding. I gave them things and they carried my children just of a sudden I heard that, we are taking marital clothes (lefe)to their house and I had to follow them. They brought the lefe to my house before taking it there and now her mother (lady married to my husband) promised to send me out of that house. At the same time my mother in law puts pressures on me. So also the lady`s mother claiming to push me out of the house. Every day I receive new pressure from mother in law, her mother my mate and her sisters. Day before yesterday they abused me seriously. Now that I am talking to you I am thinking of my children as the gate can be closed disallowing them to enter the house after coming back from the school. They will be moving around unnecessarily. I used to carry her child making him as my biological son and he trust me. But my 3 kids 2 female and 1 male;they can be knocking the gate but she will forget about them. It is not my children that have infertility but still receive the consequences. I even wanted to go back home now,because of those children,they maybe knocking and the gate will not be opened to them and if they stay outside they can be spoiled by men. Because of her trouble and that of her relatives when I went for pilgrimage, because her big big male brothers use to inter our house since she is living in their area. I called that old woman who is a relative to my husband to care for my children. So why all these? One must have some disturbances and has every reason to be angry. Looking at our situation (poverty) in the past and now they want take you out of the house!! How will you be happy? Now as I am talking to you I can cry on your presence. Even I am praying I use to cry. Look at his initial state? He spent years no single girl agree to marry him;my parent engaged me with someone at that time I said I love no body beside him as my teacher in Islamic school. He said in our class that, he proposed ladies at about 4 times but they all rejected him because he had no money. So I said I love him like that. Many men came to propose me but I said no he was the one I will marry to. To my surprise his mother will show me hatred with no reasons. It was her son I chose and left my choice and it was not because I don’t love the other men. Now his mummy supports my mate and tries to push me out. I have a lot of disturbances. If he is to buy a shirt for my child there will be a trouble in the house. How will I be happy. Now that I came to the hospital, my mate will be angry why should I come and that will be the reason to close the gate for my children why this? I didn’t hate her but she hate me so much a small girl!!

Q. If you have such disturbances does it push you to do something?

R. Yes, it made me think of running to my parent when I understood that, things were much and he can`t control his house because of his mummy and she want me out. So I thought to carry my children and run away. I also had second thought that, he(her husband) will be anxious if he couldn’t find me and his children. I wanted to carry my children, remove my sim card and go to our town. I swear to God I wanted to run away and leave them with the one they like, but I pity him. I don’t want anything that will stimulate his anger. I will be thinking that, I left my parent talk less of husband? Why this life (tears) why should I stay in a house always crying? Sometime I will even prepared to go I then say no. since he is not in support of his mother that is why I give him excuses; I will have gone by now since his mummy doesn’t like me. He supports me and backs me on this issues. But I use to get relieve when I cry and shade tears

Q. how do you perceive life in this situation?

R. I pity anyone in this situation. I have three children but still the life is bitter to me and even my children. I have tree but I am in deep sorrow of not having more what about someone who has non?(smile) look at me now that I am talking to you I am having dizziness

Q. related to neighbours and friends did you faced some challenges?

R. yes,neighbours are terrible,look at what happened to me? Has it been is a lady I don’t know at all , I wouldn’t worry so much. I told him I will not stop him from getting married to any lady but not this one that knew my secret; he said no he already made the decision. So neighbours are not good. It also happened to one of my neighbour, where they asked him to marry their daughter but he said no

Q. how do you think people look at you?

R. They look at me as one who uses family planning to stop delivery. Because that is what they have been thinking; they have been asking me that am I using family planning I said no. One who uses family planning is that who his husband is not interested in having children. People will be telling me that I stopped having pregnancy for me to rest; that you refused to provide younger brother for Abdulwahhab(her third born). So that is how they think about me. I tell them that this thing is from God,they say no this time one can easily stop delivery if he wish to. I swear to God I never take cafenol to stop prevent pregnancy.

Q. how will you compare your position in the society before having this problem and after?

R. Before,I conceived before weaning, people have been saying why should I be doing that? A small girl you also conceived? The baby was small because we didn’t have money then. I used to fast without telling anyone. His uncle was there but couldn’t help us. They gave out food in expense of my need. If I asked of it they will say is Alhaji`s (husbad`s uncle) remnants will you eat? I collected it like that and eat (laughing)my husband was at kano then and I swear to God I never tell him this story. If he give me N50 I was lucky I collect and say thanks. His uncle was the one sent him to kano, food was there but he couldn’t give me to prepare and eat. People thought my baby was born at 7month because of her body size. If I vomited nobody will give me even paracetamol to take. I born my second child while the first one was small which causes a lot of noise from people. Saying that, why should I leave things to go like this? I was abused seriously even at ANC level by the people. Now that I am having problem they are also saying I stopped it myself; this must put me in a pressure. People don’t understand things. If you able to deliver is from God if not is also from him. I know of a lady is not up to my age but she is having 8 children; she conceive immediately after 40 days of her delivery. A lady we married together with, she is having 6 children. I would have done that too.

Q. Can you please describe how you relate with people before and after the diagnosis?

R. No differences the way I interact with them

Q. As you shared with me that,you pass through so many things which bring some psychosocial problems, Looking at all that you have shared with me, have you been using some measures to adjust?

R. No I never think of that, but I thought to leave the house for them, if I am not in I wouldn’t see all these things,that is all I thought to reduce tension on me. My second thought which prevent me from running away was pity of my husband and my children. My parent will also be anxious of where am i? Because it happened toone woman on her way to see her parent some gang of kidnapers kidnapped her children, what about me who will leave my husband`s house and try to run with them?(laughing) that is what I have been thinking and stopped me from running away. Our town is far after Gombe called balansami. We have relatives in Lagos and other places but I chose to go there because it is far I never been there before and nobody will think I run to that town. My father is living there. That is why I thought of going there to reduce this tension. If you fight with a small mate they will say she is a small and if she cheated and abused you nothing you can do. If you tell your husband he will say is a small girl and yes you know is a small girl because she is age group with my 5^th^ younger sister,so the only solution for me as I thought was to run and leave the house

Q. Now that you stopped, did you use any ways to adjust?

R. Yes I always pray “God is enough for us” and other prayers to make me adjust and get some relieve. Sometime when I remembered, sometime I can`t even sleep and I remember frequently. If a single thing happen it reminds me of all others I passed through,even if I wanted not to but I can`t. it has already in my mind because it happened since I was young it worries more and stick in mind. If I want pick something I will forget what I want to do. I developed forgetfulness. I swear to God I inter room to pick something I just forget what I am there to do. I am not old enough to develop that

Q. Can you share with me health seeking behaviours?

R. Yes we have been looking for medicine; I did a lot of ultrasounds but still nothing was said is my problem. You will be told to come next day but you come you will meet someone different not that one. If you saw this one and give you an appointment, when you come you will meet different person and it was that first one you shared your problem with. The second person will say you have to do this and that again,has it been is only one person it would have been over by now. If you saw this one,next time the other person will say I have to tell him the problem again. I feel my problem but they couldn’t say it to me. I went so many places including samaru, sokoto and Saudi Arabia. Saudi Arabia told me it was a fibroid I can take drugs and it will go but I still can`t understand things. My husband tried to be justice to him. Whatever he is told to buy he will buy. Sometime I do stop taking the medicine since there was no benefit.

R. Did you use other ways rather than that of the hospital?

Q. My husband don’t allow for traditional medicine; he can do anything for the hospital but when it comes to traditional medicine he says no. that is why I only used it ones. I stopped because my stomach swollen at that very night I took it.

Q. What are the factors that make you to come to hospital seeking for help?

R. It is because of lower abdominal problems that disallow me to be pregnant. If you touch here (lower abdomen) I will feel as if you touched my front (vagina). If I eat food everything will be down and I got some relieve. At night I will feel thirst and if I drink water it will be a problem. The first time I came to hospital was because I had miscarriage and an evacuation was done to me. Evacuation wasn’t successful because I suffered a lot

Q. Now from all that episodes after you understood that you have infertility, were you the one who chose to come to hospital or it was someone who said you should come?

R. I have been going personally but now sometime my husband use to advise me to go may be I will succeed. He has been talking to dan kura and he tells him that I should come to the hospital. That is why I come hoping to get well and be part of my husband`s family with no stress and pressure. I hope you safe journey to Ghana and all the best in your study

Q. thank you very much,I hope you will soon be well.
