# Supplementary material for: “My mother in-law forced my husband to divorce me”: Experiences of women with infertility in Zamfara State of Nigeria
Source: PLoS One. 2019 Dec 19;14(12):e0225149. doi: 10.1371/journal.pone.0225149 (PMC6922459; doi:10.1371/journal.pone.0225149)
Supplement: S3 Transcript — (DOCX) [file pone.0225149.s003.docx]

Respondent3

Age 35, grinding grains, islamiyya school, 20 years of marriage,one child,Hausa by tribe and muslim by religion

Psychological experiences

Q. Can you share with me how you felt when you were told that, you have infertility?

R. I have been going to the hospital but all prove abortive till date as of now I stopped going to the hospital.

Q. How do you feel now.

R. I believe that is God that gives child to woman and I know He will consider me one day, therefore, I have to be patient.

Q. Can you describe how you feel?

R. What I feel in my heart?

Q. Yes.

R. I only have to be patient (laughing)

Q. please can you expatiate more? Because your situation may differ from someone else so if you don’t mind I want know how you feel in person.

R. I swear to God, I was so worried when I have been going to the hospital but not succeeded in having a child.

Q. Worried?

R. Yes you see I couldn’t have any child beside the one I had previously.

Q. You said you will be worried; can you describe it for me?

R. You see if you are living with people every one give birth and I couldn’t. You see since the worries differed, but still is God that give child so one must be patience.

Q. What are those things that remind you of this situation?

R. It is when I heard that someone has delivered

Q. So when you remembered how you do you feel?

R. If I remembered I only pray to God.

Q. So please how do you feel then?

R. If I say worried? Anybody knows what it is mean to be worried (high voice).

Q. How do you perceive life in this situation?

R. My life?

Q. Yes.

R. I swear to God no matter what I feel I try to hide it. I will leave it to myself. Initially I have been thinking about my problem.

Q. So at that time what was the nature of your thinking? People have been to advising me to seek for treatment. I tried traditional medication but all proved abortive. I swear to God that is what make me depend on God now. Even if I remembered I will pray to God and my heart will be cooled that is all. Sometime I will isolate myself and sometime I will come out trying to avoid some decision.

Social experiences

Q. Can you kindly share with me life situation in your matrimonial home about the diagnosis of this problem?

R. Yes actually when they came to understand that I have a problem, his parent told him to remarry and he did so.

Q. have you had pressure from them?

R. No when they said he should get another wife I said nothing. You know some may have pressure. They will be looking at my only daughter and be telling her “batayi goshi ba or tayi bakin baya” meaning she is unlucky girl since she has no younger brothers and sisters.

Q. Are they referring to her or to you?

R. They talk to her but I think they are referring to me since she don’t know what they said.

Q. What about your husband?

R. Yes he also gave me pressure initially that he will remarry. But he tried getting some medication for me.

Q. What about your relatives?

R. They show me nothing bad only that they tried getting some traditional medicine.

Q. From your understanding of the situation, how will you compare your position in the society before and after the diagnosis?

R. As for his relatives I can`t say since we are not living together. What of when you met in a gathering? I use to overlook things when I see them. They don’t take me as nothing.

Q. Can you please describe how you relate with people before and after the diagnosis?

R. I swear I am always free in people since I tried to be patient. Initially I showed that I was in worries but later I coped

Coping strategies

Q. Looking at all that you have shared with me, have you been using some measures to adjust?

R. I only use prayer to cope.

Q. What about social support from relatives and husband?

R. Yes my relatives use to advise me. I really I appreciate their advise because it gives me some relieves. That is why sometime I contact them to talk about my problem what I want is for them to give me some references of people with similar situation who got out of it

Health seeking behaviour

Q. Can you share with me general situation regarding your seeking for help?

R. I used traditional medicine and that of hospital. We started that of traditional medicine before going to hospital.

Q. Were you asked by someone to come to the hospital or you made the decision by yourself

R. No is my husband.

Q. Anything to share with me?

R. No is only the lower abdominal pain, but I am on drugs.

Q. When are you going back to hospital?

R. (Hmmm laughing) may be.

Q. Why may be?

R. Laughing because I see it has been long am looking for treatment but yet no issues.

Q. So you gave off?

R. Yes though all blessing is from God.
