# Supplementary material for: “My mother in-law forced my husband to divorce me”: Experiences of women with infertility in Zamfara State of Nigeria
Source: PLoS One. 2019 Dec 19;14(12):e0225149. doi: 10.1371/journal.pone.0225149 (PMC6922459; doi:10.1371/journal.pone.0225149)
Supplement: S4 Transcript — (DOCX) [file pone.0225149.s004.docx]

Responednt4

Bio data

Age 30, 1 child, education –islamiyya ,colanut selling, I had one child after 10 years of my marriage,since from then nothing yet. My child died; so also my first husband died.

Psychological experiences

Q. As a married woman with this condition how have you been feeling deep in you?

R. I enjoy myself (laughing) meaning always I think about this situation in my life which put me in worries.

Q. Meaning is not that you are enjoying?

R. Yes you will never enjoy your life without child.

Q. So share with me how do you feel?

R. The way I feel in my life is that, I use to become angry and sometime cry. This is because to have a child mean to enjoy in the world considering the importance children have.

Q. What are those things that remind you of this situation?

R. Is only one thing and if I remembered I will perform ablution and pray.

Q. Are there other things when you hear or see them make you remember?

R. As for me is only one thing, that is if I wake up and see that I don’t have a child. Everyone has a child and you don’t have, so in that situation you must be sad whoever you are.

Q. Can you describe your worries?

R. Sadness is known, but when I remembered I use to pray.

Q. How do you perceive life in this situation?

R. The way I look at life in this situation is pity and need concern from others. Other peoples` concern is of great importance for someone with infertility. This is because you don’t have children to care for you. You need people to strengthen you

Q. Going back to thinking you said you do, how do you do it?

R. I lock myself in a room and be thinking because of some words uttered on me saying that you don’t have a child. You must go into the room with worries and be thinking

Social experiences

Q. Can you kindly share with me life situation in your matrimonial home about the diagnosis of this problem?

R. Because of my problem?

Q. Yes.

R. So my husband`s relatives love me and show me concern. My husband shows me some pity and sympathy because he has some children. Any time he understood that I am not in good mood, he will call and advice me and utter some cooling words to me. This actually helps me and I like him to be with me in such instances. Even at night he also utters such words to me.

Q. What about his relatives?

R. Is only one here all are far from us.

Q. From your experiences, how does society look at you?

R. They look at me as the person with no child. Since everyone has his child.

Q. Do you receive some offensive words from people?

R. Yes as I told you that they have but you don’t. and that is what I told you causing me worry and anger

Q. can you kindly tell me such words.

R. Yes at any time I have conflict with them that is what they use to say.

Q. Can you please cut such words for me?

R. They say that nobody will concern about you or compete with you since you don’t have child and we have even the grand children. I reply that they should tell God such words. That is when we have conflict but if not even if they gathered I don’t put myself among them and I will be doing my things alone, I prefer to go to school.

Q. In your house?

R. Yes.

Q. Are you many?

R. No we are only two but some other women use to come. It is those women and my neighbour that utter such words on me. My husband told me not to mind them. I don’t mind them I will be doing my things alone in my room.

Q. From your understanding of the situation, how will you compare your position in the society before and after the diagnosis?

R. My relatives and good friends sympathize with me now that I am in need of child. No children and no other things to do to get income.

Does people take you are no body?

R. No I don’t have such problem

Q. How do you relate with people, do you look at them as if they are better than you?

R. “Relaxation” I relate with them normally. But someone with child who support him cannot be compared with one who doesn’t have. I mean you will be thinking has it been I have child, I will have been helped by my child (tears)

Coping strategies

Q. Looking at all that you have shared with me, have you been using some measures to adjust?

R. I use to pray to God telling Him that is it that you don’t like me that is why you tested me with this? He should see me through and cool my heart.

Q. Any other ways? Like adoption or something else

R. I don`t accept other people`s child because I was advised by my father that I shouldn’t do that. In the sense that, you will raise the child to certain level of life, but his parent will subsequently call him back to them, which that will add more stress on you. They gave me but I rejected them.

Q. What about your husband?

R. yes as I told you I will always be calmed by him as such I like him at my side always.

Health seeking

Q. Can you share with me general situation regarding your seeking for help?

Q. Were you asked by someone to come to the hospital or you made the decision by yourself

**R.** My sister advised me to seek for help

Q. where?

R. At shabanke GRA

Q. which type of medine?

R. It Is a traditional medicine. I started taking it for two months I stopped when I understood that there was no issue.

Q. Why didn’t you come to the hospital that time?

R. I assumed hospital was only for those with pregnancy to care for them till they deliver. i never knew that hospital can give care for infertility. It was later when my sister advised me to come
