# Supplementary material for: “My mother in-law forced my husband to divorce me”: Experiences of women with infertility in Zamfara State of Nigeria
Source: PLoS One. 2019 Dec 19;14(12):e0225149. doi: 10.1371/journal.pone.0225149 (PMC6922459; doi:10.1371/journal.pone.0225149)
Supplement: S5 Transcript — (DOCX) [file pone.0225149.s005.docx]

Respondent5

I am 27years old, married 5years ago ,I sell pure water; I am muslim by religion,I am a teacher since I have my NCE.

Psychological experiences

Q. Can you share with me how you felt when you were told that, you have infertility?

R. I was so scared when I understood that, something was going wrong with me because any woman need to have a child of her own. It is your child that will be praying for you after you have died. It was said that two things help an individual after his death, 1. An existing utilizable charity 2 A child who pray for his parent.. Looking at this I was so worried when I understood I have infertility.

Q. That put you in a worries?

R. Yes I worried seriously but sometime when I remembered that, it is God that give child my worries become less.

Q. As a married woman with this condition how have you been feeling deep in you?

R. life is full with lack of happiness, because people will be saying you are filling there toilet with big stool but no issues. How will one be happy in such situation? In that case you cannot do anything to satisfy them because you can`t give birth. This is not easy at all.

Q. What reminds you of this problem?

R. When I see a child I will just feel how I wish I have my own biological son. More especially when the child does something interesting. Another thing is the issue of mocking, if you want send their child to somewhere sometime they will be saying you should send your child and they know you don’t have. Or they will be saying she did family planning even though you haven’t done it. Or you hear them saying yes is my son I born him myself this is part of mocking meaning you should go and deliver your own forgetting that it is Allah who gives child.

Q. What are your reactions when you remembered?

R. . Yes I use to respondent to your words on the way you said it to me but still I shade tears after I enter my room because of sorrow.My heart will be beating seriously.

Q. How do you perceive life in this situation?

R. It Is only to leave things to God because if you don’t have you don’t have no matter. Life is difficult from what I told you,you will know that life is really difficult

Social experiences

Q. Can you kindly share with me life situation in your matrimonial home about the diagnosis of this problem?

R. I faced a lot of offensive words because I was even told that stupid infertile. That worry me seriously.

As for my husband I don’t have problem, except for other people they will be looking at you as socially oriented and one who caused it by using family planning, despite the fact I know I didn’t do it. They will gather and be saying a lot of things that I am using family planning or I am infertile and rest. As part of my mate/competitor (her husband`s wives) actually I received bad words from them regarding a child They mock at me. Some of my neighbours we live peacefully but others make it their hobby to offer words that I am filling my husband`s toilet with stool rather than given him a child. Yes they say it that I am filling my husband`s toilet.

Q. From your experiences, how does society look at you?

R. They often look at me as woman who has craving for child and that is true since I don’t have. People show their concern about it and sympathize with me. But some as I told you they will mock at you

Q. can you compare your position in the situation before and after you are known to have infertility issue?

R. The people I relate with many of them show concern to me saying that oo! our friend still awaits gift from God. But some tell me such words I told you; they will be saying something as if they are referring to themselves, but actually they are referring to me indirectly.

Q. Can you please describe how you relate with people before and after the diagnosis?

R. I relate with them normally; I didn’t change anything. I don’t think they are better off than me because I knew is God that gives child to everyone he like to give to. And I know when my time came I will have my child and enjoy what they enjoy in children.

Q. so we are moving to coping strategies. Looking at all that you have shared with me, have you been using some measures to adjust?

R. For sure I use innalillahi wainna ilaihi rajiun (We are from God and we are goig to die and return back to him) because whatever disturbs and worry someone and he continuously utters these words God will give him/her what he want. So I pray to God to give me a child who will make me happy in life. I also go to hospital

Q. You started telling me that you have been going to the hospital, were you asked by someone to come to the hospital or you made the decision by yourself?

R. Nobody advise me at the first instance, but I heard people saying that lower abdominal pain can cause infertility and I knew I suffer from it and I looked at my situation said they are really saying the truth. So that what made me to come to hospital seeking for help before it is too late. I have been patronizing many hospitals ranging from general hospital, Dr joseph`s hospital, king fahad women and children hospital and finally came back to federal medical centre Gusau. I really patronized much hospital.

Q Are there other things beside lower abdominal pain that made you to go to hospital?

R. That is it because I was told that, it causes infertility and I spent years without an issue, so I have to trust them and go to hospital seeking for medication. I know if it is the cause by the time it is been treated I will have my child.

Q. Beside going to hospital did you used other ways?

R. I used traditional medicine for my lower abdominal pain but not to seek for pregnancy, I only sought that in the hospital.

Q As you said that, going to the hospital was your choice, what about your husband?

R. yes he agreed and even supported me

Q. didn’t you face any change from him since you spent years without an issue?

R. eeeh toh!! Honestly is only God that is perfect. Anyone who planted a seed will wish to see the it grown. Nobody will say that he don’t like child. As a woman you want give birth and a man who married you want also you to give him a child. So he felt otherwise and unhappy

Q. But has he shown that to you?

R Yes even if he showed was not pronounced

Q. Anything you want add to me?

R. Issue of infertility is a serious problem affecting women psychologically, it make them spend their life looking for way out. I use prayers especially when it is raining, you know God accept prayers while raining.

Q. Thank you very much and God bless you.

R. Ameen.
