# Supplementary material for: “My mother in-law forced my husband to divorce me”: Experiences of women with infertility in Zamfara State of Nigeria
Source: PLoS One. 2019 Dec 19;14(12):e0225149. doi: 10.1371/journal.pone.0225149 (PMC6922459; doi:10.1371/journal.pone.0225149)
Supplement: S6 Transcript — (DOCX) [file pone.0225149.s006.docx]

Respondent6

Salamun alaikum. As I explained to you that my colleague who is studying his masters degree at university of Ghana is conducting research on psychosocial experiences of women with infertility and their coping strategies.

Please can you tell me little about you?

R. I have secondary school certificate from there I couldn’t proceed. But I am still doing my Islamic education.

Q. Your age?

R. I am 30 years old

Occupation/

R. I am doing our usual internal business as married woman. I am tailoring and also sell palm oil

Q Your tribe?

R. I am hausa fulani by tribe

Q. Your religion?

R. I am a muslim

Q. Then the next thing is psychological experiences due to infertility. We want know what you passed or you are passing through due to infertility problem. Can you share with me how you felt when you were told that, you have infertility?

R. I swear to God I felt disturbed and depressed. I cried and also prayed to God. Also issue of psychological disturbances are there, because even if someone is not referring to you when he talks about a child you will feel as if he is actually referring to you. You will feel like they are showing you that you are not the one who gave birth to him.

Q is like stigma, you will be feeling as they are referring to you indirectly?

R. yes they will be accusing you indirectly; actually women need help in this aspect. Some never give birth at all, some it might stopped after one or two even the later need help because they will be wishing for more.

Q. please can you describe your disturbances and worries for me.

R. Whenever I feel that I will come in my room and cry. After that, I will perform ablution and then pray to God. Then I will try to sleep. This is how I do it to get some relieves. But actually there are disturbances and worries.

Q. So as married woman with this problem how do you feel deep in you if you remember I married for long without an issue?

R. you will feel worried because I could remember there was a time when one of my younger sister got married and then in my mind I said, now this lady will get pregnant and deliver without me getting to that stage. I was 5 years in my marital home then. That was exactly what happened. You see this is annoying. It makes me think as if I am not going to deliver and have my biological child. This is because all your sisters and age mate together with whom you got married delivered and you are still there hanging. Some of them have their pregnancy so frequent and they don’t want it. If someone said that, I will feel as if I should be like her (node head down and closed eyes)

Q. what and what reminds you of this problem?

R. Yes is when I see my mate in the home doing some things to her child. As for my mate if she beat her child I will feel how I wish I have one to beat and nobody will say anything. But if I beat someone`s child they will say I cheat him because is not mine, even if he deserved to be punished. So all these make you disturbed.

Q. Then what reminds you of this problem?

R. There was a time when a small child (noded down with eyes closed) he said he want sleep here in our house, and then he was asked to seek for my permission. He said how will I seek permission from someone who doesn`t has even a rotten baby. This is a younger brother to my mate(my husband`s second wife). No matter how I tried to avoid worries….(kept mute). I responded to him that actually there is no need to seek for my permission. He may not take it as it hurt me, but it actually hurt me and even now it hurt me when I remembered and is now at least 7 years. I swear to God I still feel it in my heart.

Q. Are there other things again?

R. Yes, like you have a problem with your mate she will put her children in the room. You see she has another way to relieve her tension and worries. But as for me I am alone, that also makes me come into the room and cry

Q. what is your reaction then?

R I will feel serious disturbances, so the only thing is to pray to God that is only when I will have some relieves. I also cry seriously. I am thinking that it was the basis of my hypertension (deep breath)

Q. so you now have hypertension?

R. yes I have it.

Q. You also don’t have family history of hypertension?

R. No it is actually due to this problem, because I always think about it and I don’t have any problem worrying me rather than infertility

Q How do you perceive life in this situation?

R. Yes life in this situation is worrisome but I actually never think of leaving this world because I always think of what have I done to God to bless me with paradise? I still think I have a lot to do to get paradise. So because of that I never think of death as solution to my problem.

Q. The next thing is social issues, which will focus on how you relate with people and vise versa. Please can you share with me life situation in your matrimonial home?

R. yes oo, life is not easy for me because my mate, rather my husband always sits me down and try to calm me given me some psychological assurances. I am the one who think of whether he is going to divorce me but he always tell me that, is God that gives child to whom he want to. So I should exercise patience.

Q Considering our culture which is in full of need for children, what are some of your experiences in relation to your relatives, husband ,his relatives and friends?

R. yes there is one of my husband`s relative, she didn’t give birth for more than 20 years. She told me that her case is easier since her husband is having only her as a wife, so nobody know whose fault is that. Unlike in your case in which you are two and the other one delivers. So this shows that is my problem. It actually hurt me and I do remember her words. And she is more than 20 years without an issue but she is telling me that.

Q. what about the side of your husband any challenges?

R. I swear there is none. He even tries to calm me down. Any time he saw me worried or angry he will come and give me some advises and I like it because it helps me to withstand the hardship of this infertility. My mate also doesn`t utter even a single offensive word to me. But some of our neighbours accuse me of doing a family planning or I did and now I am suffering the consequences.

Q. When they uttered such words how do you feel.

R. I swear it hurt me seriously, because I take it as if they don’t trust me. They think that, there is something I am doing secretly. This involved even my close friends and neighbours, they think I am using family planning or I used it before. So this hurt me more because I see it as even my close ones think otherwise. They think as I am the cause

Q. From your experiences, how does society look at you?

R. No any problem, only that sometime during discussion, they will say I am still not be able to deliver

Q. Can you tell me how you relate with people before and after you are aware of this problem?

R. the only thing is that regarding children I am very cautious, I don’t normally said I most bring them back to order in the event of wrong doings. This is because I want avoid things that might arise, since they can see it in other way round. More so my husband has been telling me to be cautious on discipline children even if their parent said i should do, because they can be hurt as well.

Q. now we are moving to coping strategies. Looking at all that you have shared with me, have you been using some measures to adjust?

R. yes, I leave my things to God because he is the only one that can grant me a child. No other thing is we go to the hospital because I don’t usually go to babalawo. Some people will say this problem is associated with angel. My mother is the only one that can support me to go to babalawo, but she always tells me to go and exercise patience. She said if I go there and get stupid child what will I do? So I buy her understandings and remained as advised.

Q Now we are moving to health seeking behaviour. Can you share with me general situation regarding your seeking for help?

R. I went to the hospital to seek for treatment

Q Were you asked by someone to come to the hospital or you made the decision by yourself

R. I swear there is none, I only prayed to God to bring into me the source of my cure; Source that will find out my problem and solve it. As I was praying my husband told me to go the hospital. I visited FMC and general hospital. I never use traditional medicine because everybody will lie and say he has the medicine which is not true

Q. Is there anything you want add based on the questions I asked you?

R. no that is all I can say

Q. thank you very much
