# Supplementary material for: “My mother in-law forced my husband to divorce me”: Experiences of women with infertility in Zamfara State of Nigeria
Source: PLoS One. 2019 Dec 19;14(12):e0225149. doi: 10.1371/journal.pone.0225149 (PMC6922459; doi:10.1371/journal.pone.0225149)
Supplement: S7 Transcript — (DOCX) [file pone.0225149.s007.docx]

Respondent7

Q. Good afternoon ma

R. good afternoon

Q. As you are aware that my colleague who is pursuing his masters at Ghana is carrying out his research on psychosocial experiences of women with infertility and their coping strategies in Zamfara. I am serving as a research assistant to him. Please tell me little about you?

R. I am 35 years old. I sell tomatos, my religion is islam and I only attend islamiyya school. I am hausa by tribe

Q. Then we are now going to next stage which is psychological experiences. Can you please share with me how you felt when you were told that you have infertility?

R. I actually had problem, because if I visited a woman after delivery I will feel as if I should come with the child. If my mate delivered, I will go in my room and cry a lot. Whenever I see someone with his child I will collect the child and be playing with him, I even wean some children just because I love children and I spent a lot looking for a child.

Q. you spent how many years of marriage now?

R. I am now 22 years of marriage

Q. As a married woman with this condition how have you been feeling deep in you?

R. I still need child and that make me worry a lot because sometime I even lose my weight. This is because I don’t eat much. I was told at the hospital that my blood pressure has risen.

Q. So you think is because of your infertility?

R. Yes because I receive some offensive words because of children. They prefer to take their children to our neighbours than to allow me carry them. These are the things that worry me much which I think are the causes of my hypertension.

Q what and what remind you of this problem?

R. I now stopped thinking of that now. But at that time whenever I saw someone with a child I remember. Or if I go to naming ceremony, that day I will definitely have fever. Because I will be thinking has it been I am the one who delivered I will have been out of this situation. Or if one of my younger sister delivered I will be thinking and hoping that child is mine

Q. As you remembered what has been your reactions?

R. I use to go to our house and rest their so that I will feel little relieved. I also hide myself in a room and cry. When I came out of the room people will see the sign of worries on my face and asked what is happening I tell them that I am not feeling fine

Q. How do you perceive life in this situation?

R. I sit down and pray I will tell the God that I didn’t offend you, you give others children to the extent of some of them don’t want deliver again but you denied me a child. I pray that you give me a child. I pray a lot

Q. Now we are going to the social experiences. Can you kindly share with me life situation in your matrimonial home about the diagnosis of this problem?

R. I received bad words due to my infertility and this come from my neighbours and my mate telling me that I am barren/infertile. They say I only fill their toilet with stool; her husband gives her food but she refused to give him a baby. There was one woman who said why will he divorce me and marry another wife; a wife that will deliver and create togetherness in your children. That hurt me and made me angry. There was a time I left my husband`s house to my father`s house because of that.

Q Considering our culture which is in full of need for children, what are some of your experiences in relation to your relatives, husband ,his relatives and friends?

R. My mate actually utters a lot of bad words to me. Even my husband sometime when my mate delivered I observe some changes from him and concentrate more on her than me. That also hurt my heart (high voice). But he stopped doing that to me afterwards. If she delivered he will give me 1/3 of what he gave to her. That is when he understood that I was not happy with that. As for his relatives I don’t receive such negative treatment from them.

Q. So how do you feel when you received such words?

R. I use to be sad and worried. I will tell them clearly that I don’t want that. But if my husband did it for me, we fight. This is because my neighbours will be saying is a joke and play but my husband we live together so when he said it he meant it. As for my mate if she uttered such words we also fight. There was time when he sent both of us to our parent`s homes.

Q From your experiences, how does society look at you?

R. In those days people look as they are better off than me, but now they only pray that I should be blessed with child. If I couldn’t get the child hear in the world let me be given in hereafter.

Q. From your understanding of the situation, how will you compare your position in the society before and after the diagnosis?

R. They will be saying I am infertile

Q. Can you please describe how you relate with people before and after the diagnosis?

R. No I didn’t change; that is how I have been doing it with them

Q. Now we are going to coping strategies. Looking at all that you have shared with me, have you been using some measures to adjust?

R. I actually received a child from my husband. She is now matured and closed to get married. The way she is closed to me is not as close as she is with her biological mother. Always I looked at her I will feel less stress because she respects me; I send her wherever I need. She has been doing well to me, especially when she reached her maturity.

Q. Now we are moving to health seeking behaviour. Can you share with me general situation regarding your seeking for help?

R. I have been going to the hospital; I did so many scannings. I suffered with lower abdominal pain then and spent 3 months without menstruation. So after the investigation they said I don’t have pregnancy. They wrote drugs for me, thereafter the menses began to come. From there I spent almost 7 months menses doesn’t come; I thought that I was pregnant. I went to the hospital again. They ordered for scanning and I did. The result showed that, I was pregnant for two months.

Q. Were you asked by someone to come to the hospital or you made the decision by yourself?

R. many people have been telling me to go to the hospital including my husband. But his involvement was after I told him that people advised that I should go to the hospital. I used also traditional medicine but that was after I went to the hospital. But my husband doesn’t like traditional medicine; he was not aware when I used it.

Q. Why did you choose to use traditional medicine?

R. This is because I have been going to the hospital but still nothing had happened. They always order to do scanning and other investigations. Until when I became tired then I dropped down all my hospital cards and results and stopped going. I made that decision based on the understanding that, pregnancy is given by God so let me wait for his blessings.

Q. After that 3months without menses and you thought that, it was a pregnancy; but it became not following investigations. How did you felt?

R. I was so angry and sad because I couldn’t eat that day. I felt so disturbed. I even said no they are lying until later I accepted that I was not pregnant.

Q. So now are you still seeking for help or not?

R. I don’t I stopped that since, it became part of me. I don’t normally seek for anything.

Q. Anything you will add to me/

R. Yes I actually don’t forget with what my husband did to me years back; he told me that I should leave his house since I can`t give him a child. I can`t forget with this this, any time we may be discussion with someone I use to tell that, my husband said I should leave his house because I can`t deliver for him!!

Q.
