# Supplementary material for: “My mother in-law forced my husband to divorce me”: Experiences of women with infertility in Zamfara State of Nigeria
Source: PLoS One. 2019 Dec 19;14(12):e0225149. doi: 10.1371/journal.pone.0225149 (PMC6922459; doi:10.1371/journal.pone.0225149)
Supplement: S8 Transcript — (DOCX) [file pone.0225149.s008.docx]

Respondent8

My name is Yakubu Lawali an MSc student from University of Ghana. I am conducting my research on psychosocial experiences of women with infertility and their coping strategies in Zamfara,Nigeria.

Q. Can you share with me little about?

R. I was born in 20^th^ 1975 in the family of 7. I am the most senior child of the family. I got married 2009 as the second wife of the family. So I spent 2 years in that family. I got pregnant 2010 then after 2 months I lost that pregnancy. then….

Q Your qualification

I have diploma in nursing education and I also have certificate in general nursing and post basic midwifery.

Q Are you working?

R. Yes I am working with state school of nursing and midwifery Zamfara state Nigeria.

Q your religion?

R. Is islam

Q And then your tribe?

R I am hausa by tribe

Psychological experiences

Q Thank you very much. We are now moving to second part which is the psychological experiences. We want know what you passed or passing through since when you have this problem. You have been to the hospital seeking for medical advice or treatment on this issue and you have undergone some investigations. Can you share with me how you felt when you were told that, you have infertility?

R. After all the thorough investigations were carried out, I found out that I have a little problem, then I found disturbed. Disturbed in the sense that, every woman she was hoping that, after her parent give birth to her, she will also bring her own grand children. I am so disturbed disturbed, later after on I, if I said I am not disturbed gaskiya (truely) may be is lying. Up till now I am speaking to you some time if I sat alone I start to think things, that my younger ones they have about 5 6 children but being me the most senior child of the family, I don’t have any.

Q. so now that is at the time of diagnosis, though you started telling how you feel currently, As a married woman with this condition how have you been feeling deep in you?

R. Deep in my heart I was disturbed emotionally, but I still struggle struggle to see that I conceive but up till now I don’t know what is the problem after that investigations, the Dr the ultrasound, hormonal investigation, also have been carried on but up till now I haven’t conceive. So in within me I am disturbed, sometime I can’t eat I cannot even able to sleep.

Q. Can you share with me how your disturbances or worries look like?

R. ehhh,() I will found angry without even a cause, some time I will isolate myself. I don’t want to talk with anybody, sometime I even shade tears.

Q. thanks you very much. Can you briefly share with me, how you perceive life in this situation

R. yeah I perceived life very difficult because I isolate myself. Sometime if I met with colleagues they started discussion about their children, I feel how I wish I am among those people that enjoy their children`s life, telling sort of nice things about their children so I look at myself and say is that how I will come to the world and go back without having a child? So I was disturbed.

Q. thanks you very much. So now we are now going to the social aspect

Social experiences

You told me that you are married?

R. yes

Q. Can you kindly share with me life situation in your matrimonial home about the diagnosis of this problem?

R. yeah, I told you that I got married 2009. After staying about 2 yeas I divorced. I conceived 2010 but the pregnancy did not stay. My first experience in my matrimonial home (frown and hiss) I can`t say that I am happy because I was not staying with my husband. Yeah, I was with my husband but staying in distance some time I will stay about four month five month before he come in, so I think the problem it was due to distance, but when the things are not moving fine, the situation was just divorce. Then I got the second married 2012

Q sorry I don’t want cut you down, but you said you are being divorced?

R yes

Q. Was it because of this situation?

R. No because my husband accepted it with good faith, he was even the one giving me psychological support so that I should not disturb. It is just something that is inevitable

Q I just wanted to know if it was due to infertility, so you started saying something

R. yes I got married 2012 I am still the second wife now in the family we are three. So when I married him he has his children, up till now I didn’t conceive. So, so sometime in the matrimonial home I will be looking very sad, up to become disturbed and then he council me and even relate it to my own religion and said I that I should collect it with good faith. If God said this is my faith I should accept it. So I don’t have problem with my husband regard to my infertility.

Q. So what about your relatives and that of your husband?

R. yes my relative? Especially my mother she was so disturbed, everyday she continue disturbing may be telling that I should go to other people to seek for advise to ,to seek for medical advice. I have undergone all the investigation. But her own fear is that, I got everything in net, but is only one thing that she didn’t get from me that is her grand children. But all of my sisters and brothers are also disturbed.

Q. what about relationship with your husband?

R. My husband`s relatives more especially my mother in-law, she was so disturbed, every day when I go and visit her is the issue of one thing, that is praying for God that I have give a birth in that family. She even compare me with one of her daughter, she also has this infertility problem. So that is her disturbance that God should give me my own child in the family.

Q. what about the other husband`s relatives?

R. The only challenge I face was after I got out of the house, but he didn’t come to me face to face and discuss, but is just a side talks that is one of the problem that. His family was mock at him that he married me up to 2-3 years I didn’t conceived. So that is the reason why he said that, may be he didn’t justice to me, we are not staying I the same environment, maybe he contributed to my infertility. So he see that let us just divorce after the marriage. So about his family I can say that, I have a good relationship with his family only that I cannot say that is 100% perfect

Q. Ok, so as for the physical contact you didn’t face any problem with them?

R. No there is no any physical problem with them only the mother she will be complaining, we are not leaving in one place not in one state, but everyday her own problem is that, she continue asking me how far have I conceive? all sorts of things. I don’t know may be is disturbing her psychologically or she just wants seeing her grand children.

Q ok thank you very much. Now from your own experience how does society look at you because of this situation?

R. Some time society look at me as if I have some hidden problem may be I don’t want conceive. There was a night we went for wedding ceremony, is a matrimonial something that we meet with other relatives. They will say that if I am using any type of family planning I should stop it. I said I am not using anything, that it is me that don’t want the pregnancy. I said how will I say that, if don’t want the pregnancy I could not even get married for the first place is only God. The they will say that, that is the reason why is good for a woman to marry early, but I went and force myself, may be my mother or family support me I should continue going to school. So maybe they are thinking that the period that I took the period that I have to get married may be I exhausted that period, may be that is the cause of my infertility. So some will sympathize with me; some are thinking that I was the person that don’t want conceive. Since I am health personnel I have something that I am doing to prevent myself from getting pregnant.

Q. With that do they look at you as someone of no importance or what?

R. yes truly sometime what they are saying is that, ehmm, what they are thinking after my death I don’t have anybody to pray for me. Everybody is hoping that, after his death his children will be praying for him that even the relatives will pray is not up to like is the children going to pray. So sometime I feel so bad I isolate myself, even I am invited for any occasion I don’t normally attend

Q so you don’t normally attend?

R. Just to avoid any embarrassment, is not embarrassment but I will feel in my own part is embarrassment because may be that is why I wanted to isolate myself. I don’t want attend a place that now they will discuss issues that is going to disturb me. So is not all the occasion that I normally attend except that became undermost for me to attend. One of my families may be a friend that I will feel bad if I didn’t attend. So that is all.

Q. Thank you very much. Ma From your understanding of the situation, how will you compare your position in the society before and after the diagnosis? Because you told me you think it was due to distance you are having with your husband but later you realized that it was not. Can you compare the two periods.

R. Before even myself I don’t think I will be in this position, because normally one of the sign what that make a woman to say is fertile is her monthly menses. So I don’t have any doubt thinking that I will not be conceived up to date. When I was married I married proudly but after nine months or a year I will get pregnant and have my baby. So when things have changed I began to doubt myself. Because there was time that I just miss a month my menses ceased, when I was in kad poly. So I was so scared and I met my doctor and she said may be that is the change of environment so there is no any problem. So I have 100% that when I got married I will conceive and have my baby like any other woman. So when I got married I spent about a year, since 2009 – 2010 I was thinking it was the distance I hope that i can conceive. I have a problem I went to the hospital that I am going to that pregnancy. Since from then I was thinking that it was distance, because he was schooling then in zaria. I was thinking that when I came in during my ovulation things are going to be put in position. It was just something that is inevitable that our marriage ended 2011. So presently my position in the society, in fact there is no single day that I will not think of situation that I am facing. Now I will just think that, is that how life that is going to continue with me? is that how I am going to end? So I counsel myself that is a destiny that I should accept it with a good faith. But I will be very bad when I meet colleagues and friends they discuss about their children. Sometime they will just look at me again they are just sympathized with me up to now what is the problem. So if I said I am not disturbed more especially when I meet my parent they will disturb again up till now I haven`t conceive what is the problem. At the end I will sit down and said that is the destiny that if I faith it as a good muslim, that anything that God traces his servant has to collect it with good faith whether it is good or bad destiny 9 have to accept it. If God said that I am going to have a child, I am not losing hope up till now. If God say I am going to have I will have and if God say that is how I am going to end my life and go back to him I will accept it with good faith.

Q. thanks you. Though you have talked about it,I wanted to ask your relationship with the society before being aware that you have this problem and after. Because I remembered you said that you don’t normally attend gathering, is there anything you will add to me?

R. ehmmm, the reason I don’t want attend ceremony or something and I isolate myself, I don’t want to receive a word that I cannot control myself. So sometime we that are facing infertility is just a slight thing that will be related to you, that discussion is related to you even though they are not referring to you directly, you will feel that they are referring to you. In fact, they even said that we so aggressive something that we don’t need to react with we react. So that is the perception, some time they even said that we are inhuman. if you wanted to make a correction for the way they train their children they will said that, is because you don’t experience the pain how mother undergone before getting the children.

Q Did you faced any challenges in the event of conflict between you and somebody may be in terms of words or other ways?

R. Yes is colleague, something had happened in this office, I said you people suppose not to have said so, that if you are going to rear children rear them in such a way that people will benefit . so she said, I will never understand because I don’t have a child. If I have a child I couldn’t have made that statement that I couldn’t understand. So I said even I don’t have a child, my relatives have them. The second event again something a noise came from outside they were so scared , I said why are you so scared then one said ai gwamma ke(you have less concern )so I said I understand where you are hidden now even though I was harmed there is no problem since I don’t have anybody that I will leave behind. So you that have children if you died you have someone you will leave behind. So this is how I interpreted it. So that is why I avoid myself, sometime even if we met in the office entry to control myself, I try to avoid myself in the conversation I don’t anybody to say a word that is going to affect me and I cannot tolerate may be it will lead to another conflict

Q. do you face any social isolation from people?

R. yes there is some time when they are going to do something that has to do with children, they will say that, leave her she don’t have experience to that she will not give us the experience that we required

Q. now we are going to second to the last stage. Looking at all that you have shared with me, have you been using some measures to adjust?

R. One I have to sit myself down and counsel myself, sometime if you share a problem with people you are adding more problem to yourself. Yeah some time I avoid myself from sharing my problem with people. I keep it within myself

Q. so that are some of the strategies you use?

R. yeah I keep it within myself, I don’t even bring my infertility problem with anybody. Because I discussed with my Dr he just say that I should wait for the time. So by the time I shared my problem with people they will start to(long silence and phone rang) because I undergone investigations and nothing was identified as the problem. So I avoid sharing my problem with people because they will start telling you word. Now like one day I discussed with one of my friend, I was so surprise she accusing that it is because of my friend (step wife). Is aid no bye the way we are not even leaving in the same environment with them and this is not my first marriage. So If you start sharing your problem with people, there are people that will give you way out and others the other way round so that when you implicate yourself you find it difficult. So avoid sharing my problem with any one if God said I will have a child I will, so I counsel myself.

Q. so you said you don’t share your problem with any one, what about your husband

R. I sit with my husband but he is not a medical personal he wouldn’t understand my problem. There was time when we had a discussion with him, so can`t say that he is 100% supportive, because sometime if I follow his word we are going to have a problem with him. Sometime he will say that this is the problem with health personal, that why can`t you try another way and I said as how? He said let us go to the local house and I said I can`t involve myself in such kind of things since this is the only place where they can be able to investigate my problem and since they have investigated I don’t have to go may be they will bring a problem that may be I don’t have it. May be they are going to bring a problem between you and me. So he got angry for 2 days we were not talking with each other that even though he wanted to assist me I am not even ready to assist myself. I am also deceived with my profession. So there was a time again, I went to a friend she gave a prayers that I have to undergo. The prayer is for my husband to do and I meet him and told him that this is a prayer for one to conceive. So he said that, am I the person with infertility? I have my children. So I said actually you have your own children if I said you are the one I am not doing justice to you. Because we married and even after me he married another one and presently she is pregnant. If they told me that is women that will do it I will not even contact you will do it silently. So just felt that is you that I can bring it to. So after two days I avoid myself I don’t have any conversation with him. So when I went to his mother she said that, I should try some different then I told her that eh mama (Yes) I collected a prayer from somebody that it is the husband that is going to perform that prayer at least for 3 days, so I was surprise that when I told him nine banhaihuwa? (am I the infertile person?). So she said is that what he said I said yes. So don’t know whether she talked to him then later he came back and meet me that where is the prayer and I said leave it. I even make it that if I am the person that will open my mouth again and find any assistance in regard to my infertility I will never discuss it with you. So that is how I keep myself. Sometime he will just tell me that he went to so so person that he has a herb. After 3 months he said there is a person that is going to help us that I have to undergo some sacrifice. I said if that is the only thing I will do to have a child I preferred to stay the way I am than to go and divert myself to another practice then God will say that let me leave you and go ahead and may give me a child in life that I am going to disturbed not only me even the society will cry with that child. Then he went and tell his mother that, he is ready to assist me but I am not ready to assist myself. Then I told her mama the way that he wanted to do I will not do it. I have said that I have undergone several investigation I don’t have a problem and now I said if this infertility is going to have a problem to him let us just separate. If he felt that is feeding me is clothing me is not benefit anything from meso if that should be the cause to his life or to frustrate my life, let us just divorce ourselves. Then she said that it don’t lead to that extend. If he said so, what of his younger sister? His younger sister spent about 10 years without even having a child not even a day that she has conceived. But your own you a hope that you are going to conceive. You are not his only wife talk less of other people will be looking at him and be mucking at him. I said eh tog I met this person and another person meet me. The person that met me has conceived will I say is his problem? He is not ready to support me psychologically let him leave the way I am that is how I counsel myself. I don’t discuss any issue with him regard to my infertility he just at me and I look at him

Q Are there other ways you use to adjust to your infertility?

R. yes, i use diversional therapy, some time i will listen to islamic cassette, sometime i will read holy Quran, some time i will listen to preaching, i watch television just to divert my attention. because some time even though i was sitting with you think you are discussing with me not knowing that, i am outside minded, thinking of the situation i am facing. When my sister` children visit me or spend a weekend with me I feel that how I wish they are my children

Q. some women may think of adopting other children how it is in your situation?

R. I don’t adopt children but I have children that are staying with me, they are about 3. I keep them so that I feel at home so that I cannot feel any problem. Presently they are al, under me, I take their responsibilities. I put them in the school, all are under my care

Q. k some women use to spend on themselves thinking that they don’t have children to inherit them. How is the situation around you?

R. for me if I said I spend only on myself I become selfish. I have my mother, I am the most senior in the family our father become late,I have ageing mother. What of my ageing mother what of my brothers? Is only two that are working so I have to support them. What of the children I said I am going to stay with them. So if I said I will not spend on them why will they accept to stay with me. I just now put myself just take it as if they are my own blood children. But whatever you do to them sometime that they will show. Sometime if they show that I feel disturbed. We are continuing may be when they became matured they can be able to relax. Everything they need I provide to them. There was a time when the male one when he went to visit his father, I am so surprise may be the school asked him to get something. So when his father came he told him that he need this he need this. So when he came with those things I asked him where you get all those things from. He said is his father. I said so you can`t tell me , so go back with these things to your father and keep them, so the father has to collect them. So sometime it pains me, now he is showing me that, I am not his biological parent. So sometime I have to overlook it. I just wish they are my biological children they wil, not treat me in such way

Q. finally we are going to health seeking help

R. I normally visit my Dr who is obstetric and gynaecologist. He counsel me that I don’t have to disturbed that I should just wait for a time. Sometime if he give me appointment I will just ignore it, so I say what is the essence of going since is just a one word,no any word that is changing,some time they have to even call me that they didn’t see me. I will just say Dr I am tired. Sometime he will say that he want see his own chief in the working place or ask a colleque regarding the infertility issue so that if they have the idea they will call me. I will tell him if he has any infertility CD plate he should call me so that I can be able to have it. So if I am sick again I don’t want go to the hospital, sometime I will just take my drugs and that is because if you go to the hospital, they will start asking you questions. Have you married? Have you get children? How is… so it will make you recal event that I wanted to take it out of my mind. So I go to the hospital if it is under most I have to go, sometime I ignore it

Q. so it is because of those question you don’t go to the hospital?

R. yes because is not changing. Some time if I complain they will say is time if I insisted the will order me to go and do scanning

Q were you asked by someone to go to the hospital or you just feel is necessary for you to go?

R . When I lost my pregnancy I don’t expect that I am not going to have second pregnancy so iu just ignore it. So I just see it may be is time. So I was asking some women that after abortion is there any problem that will make someone not to conceive again? Then they say no. so I asked why it is me?. So when it is 2months 3 months I didn’t conceive I asked a friend, since my abortion I haven’t conceived. She said I should take clomid. I said aa!! Does it reach to that extent? So when I asked my first husband I he said I should buy a clomid. I said under whose supervision? So decided to meet dr consultant gynaecology. He placed me on investigations and said that there is no problems. He placed me on clomid that was not effective because the rule and compliance as I told you earlier me and my ffirst husband we were not together so there are certain days that you can continue taking then the drugs by the time you are in ovulation period you should be with your husband. He gave me specific date that I should take my drugs and meet my husband. So I calculated on these days my husband is not even around. So I met him and say Dr the day you said I should meet with my husband, I calculated that my husband is not even aroud. He said that if there is any way I can call him so that he come back. So when I called him he said that, that very day he has exam,he has so so. I said that what is the essence of taking the clomid then I stopped. So even my present Dr asked me to take clomid. So I know my husband 100% is not supporting me on any medical something then my mate will also may not understand me to assist me. After taking drugs they said that,this drugs? Is just that he is not ready to cooperate,but when I discussed with my mother inlaw she ok she will discuss with him so practice it but uptill now nothing

Q. Have you tried using other ways of treatment before coming to the hospital?

R. Since I am health personnel, I believe that the only place I can get assistance is to fine my medical status to see what is the problem I don’t look any assistance. So it is later that I have been going to the hospital I don’t have any problem. Later on since I have a mother she tried to get other means of help so all is in vein.

Q. Other ways?

R. Yes other ways may be Islamic herbs, so any time we went is this usman something medicine store. So we met the Dr, he also asked me, because he is join it into two both orthodox medicine and herbs,s o he place me on investigations, I have done it and said that I should continue taking this herbs. He missed some herbs with some oil I also take it up till now I couldn’t see anything I also stopped going to him
