# Supplementary material for: “My mother in-law forced my husband to divorce me”: Experiences of women with infertility in Zamfara State of Nigeria
Source: PLoS One. 2019 Dec 19;14(12):e0225149. doi: 10.1371/journal.pone.0225149 (PMC6922459; doi:10.1371/journal.pone.0225149)
Supplement: S9 Transcript — (DOCX) [file pone.0225149.s009.docx]

Respondent9

As i explained to you,my name is Yakubu Lawali studying at university of Ghana. Studying MCH.I am conducting a research on psychosocial experiences of women with infertility and their coping strategies in Zamfara. Can you please tell me little about you?

R. I spent 11 years of marriage;27 years all in the world, but I never miss my menses which normally make one to feel as if she is pregnant, so I never miss it truly. I am married not doing any business. I am hausa by tribe. I started islamiyya school but I stopped later.

Q. we are now moving to psychological experiences. Can you share with me how you felt when you were told that, you have infertility?

R. yes after I went to the hospital they gave me some test to do but my husband said he doesn’t have the money and I also don’t have. So I kept it aside. When I got the money I told him to give me the test request he said it was with his boss. When he asked him he said he lost it. This was the reason why I said I have to change the hospital since I couldn’t do what they asked me to do. More so that Dr was a good one since I was told that he goes to India for his work. So that was the reason why I changed hospital but I spent some time before coming to this hospital.

Q.So from the time you knew that, you have this problem what have been your feelings till date?

R. I feel bad and unhappy. I shaded tears a lot and also thank God for that.

Q. you said you feel sad?

R. yes I feel sad seriously

Q. So can you describe it for me?

R. it makes me feel will I be able to get pregnant or not/ sometime I even think of creating conflict with my husband for him to divorce me may be is with him that I couldn’t be able to get pregnant. With thinking that, i may get it somewhere.

Q. Is he having another wife?

R. yes he married her just yesterday

Q. you said your sadness makes you cry, does it make you isolate yourself from people or affect your interaction with them?

R. Actually it makes me isolate myself, I don`t interact with people much. Even if I do not with happiness I am the only one who knows what I feel at that time. I become more angry when I am about to do menses. But after the menses it reduces.

Q. What are normally your thoughts before and after menses?

R. Every time I finished menses I will start thinking whether next time I will be able to be pregnant or not. It became part of me after every menses that will be my thoughts.

Q. What about your mate has delivered?

R. Yes

Q. You told me that you think a lot about your condition, what is the situation during and before you meet with your husband?

R. I think a lot before and even after we met. I will be thinking while I get pregnant during this inter course or not. Equally the same after we finished I think the same. Then I pray and leave things to God.

Q. haven said that you think a lot before and after you met with your husband does that thoughts reduces your sexual satisfaction when you compare it with years back when you don’t have such thoughts?

R. (quit)Yes it reduces, because sometime I will lack interest in that affairs simply because I saw the result of the fast period which ended without conceiving. Sometime I will feel even this one may be the same. But sometime I will feel I am going to be pregnant after we mate.

Q. What reminds you of this problem?

R. No there is not,what I am in is the infertility issue

Q. Yes I am talking about infertility,you said you become angry when you remembered. So what reminds you?

R. Actually when I remember the issue of death. Because if you died without a child nobody will pray for you except your parent and relatives. As for relatives some of them may forget about you easily. If you don’t have a child people will forget about you they remember you only during discussion. They will say Allahu akbar(Allah is great) so so person. But if you have a child the moment they see him they will remember you and pray for you in addition to his prayers.

Q. Anything else?

R. NO

Q. How do you perceive life in this situation?

R. Toh I will be thinking maybe I am not going to have a child, but I will also say there are others with 20 years of infertility but they delivered. So I will say I may also get my own when it is time. So that is how in feel.

Q. When you remembered what will be your situation if you become angry/

R. When I am in that situation i react negatively to people following small thing. Or I will tell them please leave me alone for now.

Q. Can you kindly share with me life situation in your matrimonial home about the diagnosis of this problem?

R. I faced nothing bad. I don’t have any problem. Just that my mate mocked at me oneday I told her that remember is God that give child to people.

Q. Can you please what happened?

R. It was one day she is going for ANC she asked of money,she said I should give her N1300. I said but he left N1000 for me to give you but you can borrow N300 when he came back you collect and return to the owner. She said hmmm some people envy what she is going to do. I said what did just said? She repeated that,already I am jelous of her pregnancy. I said no since the first day you conceived I never show you anything. If I have the money I can give you. When he came back I told him. He said to her she made a mistake because the way I care for her he didn’t do that for her. So I think she was only one who tried to mock at me.

Q. From your experiences, how does society look at you?

R. They look at me as one who deserved to receive pity. They also pray for me. They use to say this one who came after me gave birth but I am still hanging.

Q. From your understanding of the situation, how will you compare your position in the society before and after the diagnosis?

R. No things are normal, no any different

Q. Can you please describe how you relate with people before and after the diagnosis?

R. No I relate with them as I used to no any change

Q. Looking at all that you have shared with me, have you been using some measures to adjust?

R. No I only pray to God and I know he will give me child

Q. You never adopt a child?

R. I took one girl from my parent`s home. She is staying with me currently

Q. How do you look at her?

R. I look at her as if I am the one who gave birth to her and when I look at her I feel happy.

Q. What about your husband do you seek his assistance?

R. Yes my husband when he met me sad,he will try to calm me and tell me I myself is because I am a man but I feel the same. He continues doing that.

Q. Were you asked by someone to come to the hospital or you made the decision by yourself?

R. I am advised to go. Some advised me to look for traditional medicine but some said I should go to the hospital. Finally I chose to come to the hospital.

Q. You received drugs from hospital?

R. Yes they gave me a drug to take and my husband should be having affairs with me for five days. I told them I am not alone; they said I can request that from her or she borrow for me.at that time I was doing menses I told the lady who took me to the hospital. She said I should give my mate those days after all you are useless to him. I told her I will explain to her and I requested that from her. He should stay with her for five days afterwards immediately I finished menses he will come to me and she agreed. So wasn’t be able to procure the drugs and that is how it goes. From that time I continue using traditional medicine. I used it a lot.
