# Supplementary material for: “My mother in-law forced my husband to divorce me”: Experiences of women with infertility in Zamfara State of Nigeria
Source: PLoS One. 2019 Dec 19;14(12):e0225149. doi: 10.1371/journal.pone.0225149 (PMC6922459; doi:10.1371/journal.pone.0225149)
Supplement: S10 Transcript — (DOCX) [file pone.0225149.s010.docx]

Respondent10

My name is Yakubu Lawali

Please tell me little about you?

R. I sell provisions, I am 45 years old. I study arabiyya,I have junior secondary school certificate,I have one daughter. We are two to our husband.

Q. Can you share with me how you felt when you were told that, you have infertility?

R. I am disturbed because I want be training children and I don’t have them. My husband married another lady and gave birth for him. I gave birth to my daughter with another man.

Q. what does your thought brings to you?

R. some time I feel like to go somewhere may be I will be able to get some more with another guy. I do think that has it been I didn’t came back for him I would have get some children, but some time I will say no let me continue looking for treatment of my problem may be if I got cured I will be able to deliver.

Q what are the things that reminds you of this situation?

R. I told you that, if I look at my daughter I will say has it been I am with her father I will have gave her sisters but now she doesn’t have. This hurt her. It hurt her because her friends hold their sisters` hands while going to school but she couldn’t because she doesn’t have a sister. It really hurts her. So her disturbances make me remember that I need more of them and that really hurt me. I will be thinking that had it been I am with her father may be I would have get sisters for her.

Q. How do you perceive life in this situation?

R. Despite disturbances one must be patience. God love those who exercise patience when tested

Q. How do you see people with more children?

R. we are the same just them by looking at what they have of children I know they are blessed more than me with number of children. But as for quality no one knows as a single daughter of mine can be of great quality for me

Q. Can you share with me the situation in your matrimonial home?

R. I don’t have problem, because he takes care of me, give me everything I need. Equally the same my neighbours their children are like mine, sometime what I do with their children I may not do it with my own daughter.

Q. what about his relatives?

R. Before I gave birth, they put pressure on me that I am infertile to the extent at which we they force him to divorce me. They brought many girls for him to marry and he said no, until they achieved their aim. I got my daughter after marrying another person. That is when they realized that, things are not the way they think. After I came back to my first husband`s house his wife got pregnant without him knowing. I told him that you are about to have a child in this house and he was like not agreed. So when she (the second wife) delivered I faced challenges with his relatives showing me that my competitor gave birth for him and I didn’t. It reached to the extent we fought. From the pregnancy of my competitor (second wife) to delivery and to the death of the baby I faced a lot of challenges with them. They showed me that I cannot do some things in the house since I am not being able to give them a child. But later things became the way they suppose to be.

Q. Are they uttering some words on you or what?

R. Yes they were telling me that, another lady came and gave them child but I didn’t. In fact even my husband I faced some problems with him because he showed me that, she gave birth for him unlike me and rest of things.

Q. You separated with your second husband, was it because of infertility issue or something else?

R. No it was because of usual misunderstanding in life.

Q. So you said it has been hurting you because of this problem, have you used some ways to adjust?

R. I perform ablution prostrate to God and pray for way out

Q. Can you share with me ways you followed to see that you have more children?

R. I only came to the hospital and I do follow what I am advised by my Dr in terms of my treatment?

Q. Were you asked by someone to come to the hospital or youmade the decision by youself?

R. It was me that think I should come and seek for treatment. Considering the pressure put on me by my husband`s relatives.
