# Supplementary material for: “My mother in-law forced my husband to divorce me”: Experiences of women with infertility in Zamfara State of Nigeria
Source: PLoS One. 2019 Dec 19;14(12):e0225149. doi: 10.1371/journal.pone.0225149 (PMC6922459; doi:10.1371/journal.pone.0225149)
Supplement: S11 Transcript — (DOCX) [file pone.0225149.s011.docx]

Respondent11

My name is Yakubu Lawali an MSc student at University of Ghana,Legon. As I explained to you the questions are arrange based on headings of enquiry. Can you please tell me little about you?

R. Iam 27 years old,hausa by tribe,attended islamiyya school,married for the past ten years and I delivered ones after 6years of marriage. I am living with a mate. Initially I was alone but he married her later

Q. Now we are moving to psychological experiences. Can you share with me how you felt when you were told that, you have infertility?

R. I was disturbed initially but my parent cooled me down with their lovely words. Two of my sisters are also having the same problem. One of them spent five years of marriage but delivered ones and that is after she has been going to the hospital for treatment. The other one is still going to hospital; she spent eight yes of marriage but still not even an issue.

Q. All these disturbed you?

R. yes because my husband`s relatives use to come and be saying a lot of things. They mock at me and finally made him to remarry.

Q.They mock at you

R. yes they did that and finally made him to marry another lady

Q. What type of mocking?

R. They will be saying I am putting weight but their brother is reducing weight and I didn’t give anything rather that eat and give out stool

Q As a married woman with this condition how have you been feeling deep in you?

R. I need children and no matter what their number I will hold and provide proper upbringing for them.

Q. Now that they are not as many as you desired how do you feel?

R. I initially disturbed but now I am not

Q. Before what type of feeling have you had?

R. Yes when I go out of the house I feel relieved, this is because I thought about it when I am alone

Q. When you go out you feel relieved?

R. yes it will reduce

Q. how does it make you feel?

R. It just brings so many thoughts in my mind

Q. If you don’t mind can you share with me the nature of those thoughts?

R. I stopped having those disturbances

Q. Yes I am referring to previous situation

R. (mute a while) I can`t remember now

Q. it brings some thoughts for you telling you to do this and that, can you remember some of them?

R. My husband never show me anything, but his relatives because even after his second marriage he show no differences between us

Q. What reminds you of this situation?

R. Yes they did an ultrasound for me thereafter they said the problem it was in my womb so they did an operation to me. So after a year of the surgery I delivered

Q. I am trying to know what are the things reminding you that you need more children?

R. I initially decided not to come back to the hospital but my mother force me to do so by liaison with my elder brothers. This is because Dr requested for my file when I was pregnant It was nowhere to be found so now she said I should come and change another one. They ordered for ultrasound just like the one I did before because everything has come back to zero level, it was new card

Q. What are some of the things that make you remember you want deliver again?

R. I just need more children, because since she was small I wanted to be pregnant again even before I weaned her. She is now four years of age. I have been thinking that, am I going to repeat same 6 years as in the case of first child? I told one of my sibling that don’t you think I will another six years again? Because this is the fourth year nothing yet. I don’t even conceive talk les of saying I have abortion

Q. When you remembered how do you feel?

R. At that time I cried

Q. What made you cried?

R. I cried because I don’t have a child and People who mock at me put pressure in my heart

Q. like who and who?>

R. my husband`s relatives

Q. Is this that they utter some words on you or do something to you?

R. they utter offensive words on me because his mother even said if not because we have relation and that the house is not wide enough she will have ask him to divorce me and marry another wife

Q. you said his relatives utter bad words on you can you share with me such words?

R. They will be abusing me indirectly pretending they are talking to themselves. Sometime I can`t even interpret some is when I tell someone then I know the meaning.

Q. Can you share with me such words?

R. I can`t remember now. But they understood the reality and stopped

Q Regarding your husband what is the situation?’

R. He never show me anything due to my condition because he had been going to villages seeking for traditional medicine for me and if I go to the hospital whatever is written he use to buy. When they said they were going to operate me I told him that, if they were going to cut me I wouldn’t agree. He tried to convince me to the extent that he reported to my elders. They came and talked to me telling me that do I want die without children? They said do I want be like my aunty who delivered ones and the child died. She(her aunty) like children much. So it took two months to convince me. So I went back to the hospital Dr said what happen? Didn’t we book you for surgery I said yes it was because my husband travelled that is why I couldn’t come. So they wrote and give me a paper. They operated me after 2 days and I spent five days in the hospital then they discharged me. The issue is it was not all that knew that I was coming to the hospital even among my brothers. They knew only when it reached to the level of surgery. Only that my sisters knew because they have been telling me why shouldn’t I go to the hospital?

Q. From your understanding of the situation, how will you compare your position in the society before and after the diagnosis?

R. I wonder women who use family planning because if God will make me like them I will never use it except if my husband put pressure on me

Q. So those women do look at them as superior to you or how do you look at the situation?

R. There is no different among people from the side of God. I don’t know if they will look at it that way because they have many children. Some of them can even be mocking at you. For instance, there was a lady I knocked her child and she said it was because I am infertile that is why. I just told her that I heard.

Q. How do people relate with you after having this problem?

R. Nobody knew I have this problem, even my elder brothers and sisters who knew is not everything I use to tell them

Q. So looking at our culture that requires need for children and you spent some years without given birth don’t you think they will be saying you have a problem?

R. they use to say I am using family planning that is why especially my age mate who we married in the same period.

Q. What is the situation with your mate?

R. We live peacefully because conflict came from husband if he show that he loves one in dispense of the other but in our case he take us equal

Q. Has she delivered?

R. Yes she delivered ones, he is now two years of age

Q. so what did you feel when she delivered considering the fact that she met you in the house?

R. I felt nothing rather than happiness, because even if I didn’t marry him he is my brother I will be happy for him to have a child

Q. Does that reminded you of your situation or brought some thoughts to you?

R. NO it hasn’t come to me

Q. What about his relatives

R. It is now they are telling us that I and her(mate) refused to produce more children to their brother; because she still don’t conceive

**Coping strategies**

Q. Looking at all that you have shared with me, have you been using some measures to adjust?

R. Yes I use to listen to Islamic music or I use to go out of the house or if I should receive strangers that help me to forget my situation

Health seeking behavious

1. Can you share with me general situation regarding your seeking for help?
2. Yes I used traditional medicine and that of the hospital. I am thinking of my first pregnancy it was as a result of traditional medicine. Because you take the medicine for a month and then you stop for a month again to see if there is pregnancy fine, if not, then you take it for another one month. It is pap that is to be drunk. When I drunk it for the first one month I became pregnant. I gave birth after a year because when my pregnancy reached five month I bled and it was said that, it has to pay back those months(5 months) so that is why I couldn’t deliver it till after a year.
3. **Now that you** came to the hospital were you asked by someone to come to the hospital or you made the decision by yourself?
4. It was my mum that said I should come to the hospital may be is my initial problem came back again
5. Did you think of coming to the hospital or what was the situation?
6. I personally didn’t think of that because it was said that, my womb was closed and they opened it so why should I come back to hospital? That is why I didn’t think of coming back. But I thought if at all I have to I should that traditional medicine I told you about. But the issue is the woman that collected the traditional medicine for us died and she met him on the road so we can`t rich him now. He is not receiving money, after delivery then you give out charity on his behalf. If he came back after a year then you give him said amount if he didn’t come back you give it to the mosque. In my case he didn’t come back so my mummy bought mat and kettle and took them to the mosque

Q. So beside those ways have you used another ones?

R. No that is all I used

Q. What will you share with me in relation to your husband`s relative based on the pressure they put on you?

R. Now there is no pressure, initially it was ignorant if not one should know God`s planned things how he want them to be on anybody. God gives and deny as he wish. But I don’t know what make them stopped now

Q. Does your disturbances made you to be in a room and think of doing something or not?

R. No but I used to be in a room and cry whenever I remembered their words. They stopped after I gave birth to my daughter. That time my elder sister said whenever I give birth she will carry the baby and take to them and tell them here is the infertile as you said. That is the big stool you are saying it turned to something else.

Q. Anything you want share with me?

R. That is all I can say for now

Q. thank you very much.
