# Supplementary material for: “My mother in-law forced my husband to divorce me”: Experiences of women with infertility in Zamfara State of Nigeria
Source: PLoS One. 2019 Dec 19;14(12):e0225149. doi: 10.1371/journal.pone.0225149 (PMC6922459; doi:10.1371/journal.pone.0225149)
Supplement: S12 Transcript — (DOCX) [file pone.0225149.s012.docx]

Respondent12

My name is Yakubu Lawali a an MSc stuent at university of Ghana,legon. I am conducting my research on psychosocial experiences of women with infertility and their coping strategies in Zamfara.

Q. Please tell me about yourself

I am hausa by tribe, I am 23 years old, I married for the past 4 years and I don’t have any child. I have secondary school certificate. I am no doing any business

Now psychosocial experiences

Q. Can you share with me how you felt when you were told that, you have infertility?

R. I had serious disturbances and I was sad. I felt as if it was not true but when I remembered that I spent years without conceiving I began to accept. But generally it wasn’t easy for me to accept

Q. As a married woman with this condition how have you been feeling deep in you?

R. Just like any woman need to see her own child I also want see my child. It makes someone sad and lonely not even interested of communicating with people. I prefer sometime to be alone thinking of what is happening to me,what causes it and how can I get out of this situation. I use to cry sometime because it hurt me seriously(tears for long time)

Q. What reminds you of this situation?

R. You think as you will not deliver or is your husband`s problem. Or if I see someone sons or daughters or heard that someone delivered.

Q. What are normally your feelings or reactions as you remember?

R. You will feel bad and sad

Q. Does your sadness make you do something?

R. (eyes looking forward and depress)Yes I only pray (tears)

Q. How do you perceive life in this situation?

R. Life in this situation is full of sadness and unhappiness. It makes me feel as if I request for divorce.

Q. Can you kindly share with me life situation in your matrimonial home about the diagnosis of this problem?

R. (Quit and depressed and respond to question) pardon?

Q. Can you kindly share with me life situation in your matrimonial home about the diagnosis of this problem?

R. If I am sad and disturbed my husband tries to cool me down telling me that things are from God and rest. So I should exercise patience(tears)

Q. What about relationship with his relatives?

R. His relatives insult me saying that I refused to give him child they will be talking about it

Q. what are some of these words

R. Sometime they don’t need to talk about it in your present but definitely they do

Q. what about your husband?

R. my husband never says that because of this situation will marry another wife but definitely I know he will, even if he didn’t say it. Couple with my problem he will definitely do it one day (tears)

Q. what about his parent?

R. They all died

Q. From your experiences, how does society look at you?

R. they think that I used family planning, or you don’t want deliver or someone if she met you will be looking at you as one who refused to deliver and rest

Q. From your understanding of the situation, how will you compare your position in the society before and after the diagnosis?

R. They will be looking at you as not all that important because you don’t have a child but if you have children they will be respecting you as mother

Q. If you don’t have a child they will look at you with no respect?>

R. Some of them but some respect you and look at you as important

Q. Can you please describe how you relate with people before and after the diagnosis?

Q. Looking at all that you have shared with me, have you been using some measures to adjust?

R. I wake up in night pray to God to help me

Q. Can you share with me general situation regarding your seeking for help?

R. I became mobile any where I heard someone is given medicine I will go to see if I will succeed

Q. Where and where have you gone?

R. I went to the hospital since. I saw a Dr but nothing happened then I stopped going.then I came back later

Q. before you go to the hospital where have you gone?

R. like felden or traditional medicine. My husband brought it for me

Q. Were you asked by someone to come to the hospital or you made the decision by yourself

R. It was my husband who said I should go to the hospital
