# Supplementary material for: “My mother in-law forced my husband to divorce me”: Experiences of women with infertility in Zamfara State of Nigeria
Source: PLoS One. 2019 Dec 19;14(12):e0225149. doi: 10.1371/journal.pone.0225149 (PMC6922459; doi:10.1371/journal.pone.0225149)
Supplement: Attachment — Submitted filename: REPONSES TO COMMENTS.docx [file pone.0225149.s013.docx]

**REPONSES TO COMMENTS**

**09/10/2019**

**PONE-D-19-15263R1**

**“My mother in-law forced my husband to divorce me”: experiences of women with infertility in Zamfara State of Nigeria.**

**PLOS ONE**

|  | **COMMENTS** | **RESPONSES** |
| --- | --- | --- |
| 1 | Please confirm that the data provided in the 12 transcripts are completely anonymized, not potentially identifying, and approved to share publicly. | Yes, I confirm that the data provided in the 12 transcripts are completely anonymized, not potentially identifying, and approved to share publicly |
| 2 | Please confirm that all data needed to replicate all of the figures, graphs, tables, statistics, and other values are provided within the submission and Supporting Information files. | Although our paper does not include figures, graphs, tables, statistics, and other values, I confirm that all data is anonymized. |
|  |  |  |
